# Design and Synthesis of Binding Growth Factors

**DOI:** 10.3390/ijms13056053

**Published:** 2012-05-18

**Authors:** Seiichi Tada, Takashi Kitajima, Yoshihiro Ito

**Affiliations:** Nano Medical Engineering Laboratory, RIKEN Advanced Science Institute, 2-1 Hirosawa, Wako, Saitama 351-0198, Japan; E-Mails: s-tada@riken.jp (S.T.); t-kitajima@riken.jp (T.K.)

**Keywords:** growth factor, immobilization, protein engineering, tissue engineering, collagen, peptide ligation

## Abstract

Growth factors play important roles in tissue regeneration. However, because of their instability and diffusible nature, improvements in their performance would be desirable for therapeutic applications. Conferring binding affinities would be one way to improve their applicability. Here we review techniques for conjugating growth factors to polypeptides with particular affinities. Conjugation has been designed at the level of gene fusion and of polypeptide ligation. We summarize and discuss the designs and applications of binding growth factors prepared by such conjugation approaches.

## 1. Introduction

Growth factors are involved in the regulation of a variety of cellular processes and typically act as signaling molecules between cells. They promote cell proliferation, differentiation and maturation, which vary in growth factors. As a result, they play important roles in wound healing and tissue regeneration [1]. However, most growth factors act in a diffusible manner and are generally unstable in a tissue environment. This prolonged retention is considered to maintain the activity of growth factors in cells or in their environment (*i.e.*, the extracellular matrix [ECM] or artificial implant scaffolds) and to be advantageous in regenerative medicine applications, until the repair process is initiated or even completed. Thus, many attempts have been made to improve the performance of growth factors (e.g., their active period and stability). In addition, it is very important to add biofunctionality such as the regulation of cell functions to biomaterials used for artificial organs [2]. Modification of growth factors for immobilization on, or for high-affinity binding to cells or scaffold biomaterials has been performed by various researchers.

## 2. Diffusible and Nondiffusible Actions of Growth Factors

**Figure 1:**
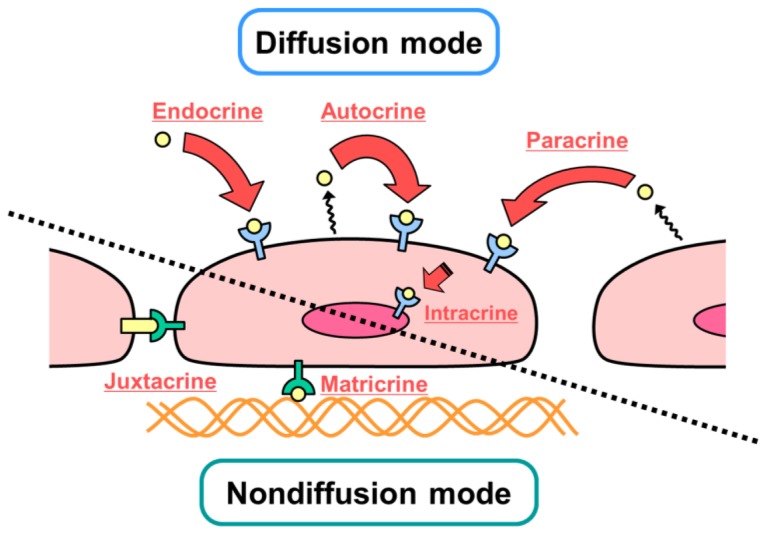
Modes of action of growth factors. Growth factors interact with their receptors in a diffusible manner (e.g., by endocrine, paracrine, autocrine and intracrine pathways) or in a nondiffusible manner (e.g., by juxtacrine and matricrine pathways). Some growth factors are known to act in both ways.

In designing growth factors for tissue engineering, regenerative medicine, and cell culture systems, it is important to consider their mechanisms of action at the cellular and molecular levels. Figure 1 summarizes the modes of action of growth factors in cells. Diffusible interactions (e.g., endocrine, paracrine, autocrine and intracrine) and nondiffusible interactions (e.g., juxtacrine and matricrine) are known to occur. It is also known that each growth factor does not necessarily have a single mechanism of action at the cellular level.

Most growth factors which act in a diffusible manner, interact with their cognate receptor on the cell membrane, and form a complex. This interaction induces phosphorylation of the receptor and triggers signal transduction in the cell. These complexes are then internalized, partially decomposed by lysosomes, and partially recycled to the cell membrane. Thus, internalization of the receptor/growth factor complexes leads to the desensitization of cells (downregulation), and to the reduction of excessive responses and overstimulation. In contrast, some growth factors are known to act in a nondiffusible manner by being present at the cell surface (juxtacrine) or by associating with specific substances, such as the ECM (matricrine). The nondiffusible mechanism was elucidated by the discovery of cell membrane-bound growth factors in the 1990s [3,4], which include heparin-binding EGF-like growth factor (HB-EGF), transforming growth factor-β (TGFβ), tumor necrosis factor-α (TNFα), colony stimulating factor 1 (CSF1), and the c-kit ligand. These growth factors are barely internalized even after binding to their receptors but exhibit long-term activity without downregulation. This point suggests the possibility of designing binding growth factors with specific activities [5].

**Figure 2:**
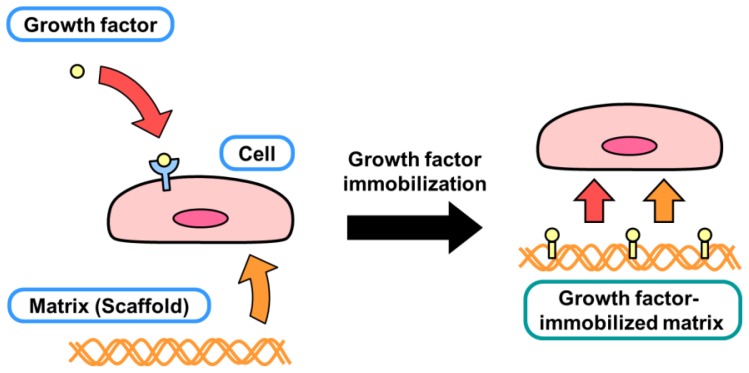
Three main factors in tissue engineering: cells, growth factors and matrices (scaffolds). The conjugation of growth factors and matrices provides a new approach for generating biofunctional substrates for regenerative medicine.

It is recognized that there are three important elements in tissue regeneration: cells, matrix and growth factors (Figure 2). Conjugating the latter two elements would enable cells to reconstitute damaged tissues stably and efficiently during regeneration. The regulation of each element is important in regenerative medicine or tissue engineering.

**Figure 3:**
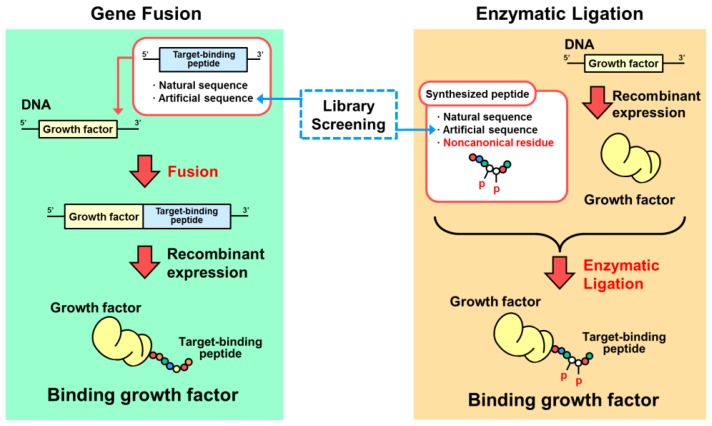
Preparation of binding growth factors by genetic fusion or enzymatic peptide ligation methods. Whereas the genetic fusion method has been used in most studies of binding growth factors, the peptide ligation approach can be used for binding peptides containing noncanonical residues such as phosphorylated amino acids.

Here we describe two ways of designing conjugated growth factors: one is genetic fusion to combine natural polypeptides and the other is peptide ligation to combine natural polypeptides (usually growth factors) and nonnatural polypeptides containing noncanonical amino acids (usually target-binding peptides) (Figure 3). The genetic approach has been developed since recombinant DNA technology arose. On the other hand, the peptide ligation method was developed to compensate the solid phase synthesis of biological macromolecules such as oligonucleotides, oligopeptides, and oligosaccharides. Because of the length of macromolecules prepared by solid phase synthesis, it was necessary to elongate them by chemical or enzymatic methods. Various methodologies have been studied using bioorthogonal chemistry [6].

## 3. Designs Based on Genetic Fusion

Much knowledge about the interactions between biological molecules and biological substrates has accumulated. Based on this knowledge, peptide sequences for binding to targets were selected and used for fusion with growth factors as listed in Table 1. These binding growth factors were produced as chimeric proteins by recombinant expression from DNA sequence encoding growth factors and target-binding peptides (Figure 3). Growth factors have been developed that bind not only to biological substrates such as collagen or cells, but also to artificial substrates such as plastics or metals. Although most of the target-binding peptides were portions of natural proteins, nonnatural polypeptide sequences were also employed.

### 3.1. Collagen-Binding Growth Factors

There are many examples of binding growth factors with collagen affinity. Because collagen is the major component of the ECM and is present in most tissues, it is a most promising target for therapeutic applications. To date, four kinds of polypeptide sequences from the collagen-binding site or domain (CBD) of different proteins have been employed to generate the collagen-binding growth factor: von Willebrand factor (vWF, 10 amino acids), bacterial collagenase (~24 kDa), human collagenase (eight amino acids), and fibronectin (~40 kDa or 27 kDa). Gene sequences encoding these polypeptide sequences and those encoding growth factor sequences were fused and expressed by recombinant techniques as single proteins.

Nimni and his colleagues produced a fusion protein of the CBD from vWF with TGFβ1 or other growth factors [7–11]. Although recombinant human collagen-binding TGFβ1 (rhTGFβ1-F2) had a lower biological activity compared with its native counterpart [7], when combined with a collagen gel, rhTGFβ1-F2 stimulated the migration and proliferation of mesenchymal stem cells in the gel, as well as their differentiation to cartilage cells [8]. Other fusion proteins of epidermal growth factor (EGF, EGF-CBD) [9], basic fibroblast growth factor (bFGF, rhbFGF-F2) [10], and bone morphogenetic protein3 (BMP3, rhBMP3-C) [11] were also reported; all showed binding affinity to collagen and an activity similar to that of the original molecule.

Nishi *et al*. produced other fusion proteins using the collagen-binding motif from bacterial collagenase fused to EGF or bFGF [12]. Both products were retained for about one week when subcutaneously injected into rats, suggesting their tight binding to collagen or other ECM components. The EGF fusion protein exhibited only 1/10 of the original EGF activity, while the bFGF fusion protein showed an activity similar to that of the native molecule. The CBD might affect the activity of the fusion protein or the binding affinity to the receptor, depending on the growth factors being fused. Brewster *et al*. employed the same CBD to generate collagen-binding bFGF (R136K-CBD) [13]; R136K is an engineered bFGF that has thrombin resistance. The binding affinity of the fusion protein was four times that of native bFGF and R136K. It is not clear if this modified molecule has any advantage over the native one, considering that bFGF itself has very high affinity to collagen.

A short polypeptide sequence of the human collagenase CBD was also employed to construct collagen-binding fusion proteins of platelet-derived growth factor BB (PDGF-BB) [14], bFGF [15], BMP2 [16], nerve growth factor (NGF) [17], brain-derived neurotrophic factor (BDNF) [18], EGF [19], VEGF (vascular endothelial growth factor) [20] and neurotrophin-3 [21]. Zhao *et al*. compared a short polypeptide sequence of vWF with that of human collagenase as the fusion partner for bFGF and found that the latter provided a higher binding affinity to collagen [15].

**Figure 4:**
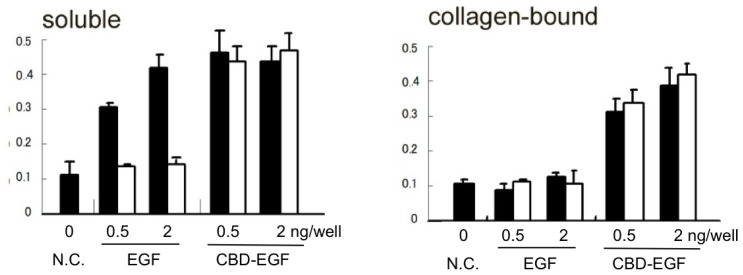
Stability of the growth-stimulating activity of collagen-binding epidermal growth factor (EGF). Fresh proteins or proteins stored at 4 °C for 30 days in culture medium (left graph) or bound to collagen-coated wells (right graph) were applied to cultures of human dermal fibroblasts (48-well plates). Filled columns, day 0; open columns, day 30. Cell growth activity was evaluated at seven days of culture using a WST1 colorimetric assay (absorbance at 450–650 nm; mean ± SD). Similar results were obtained for CBD-HGF (CBD = collagen-binding sites, HGF = hepatocyte growth factor)

We have also designed collagen-binding growth factors by a fusion of the fibronectin CBD with growth factors; CBD-EGF [22], CBD-HGF (HGF = hepatocyte growth factor) [23], CBD-VEGF [24], CBD-PDGF-BB (unpublished) and CBD-BMP4 (unpublished). These fusion proteins exhibited binding affinity to major collagen types I–V, which was not observed for the native growth factors. Moreover, they showed authentic biological activities in the collagen-bound state. These fusion proteins can be stored for longer periods than native growth factors without loss of their activity (Figure 4). The fused CBD moiety might protect the growth factor moieties from degradation or denaturation.

### 3.2. Fibrin-Binding Growth Factors

Fibrin is an abundant protein present on the surface of injured tissues. In addition, it has been studied as a material for tissue engineering scaffolds. Therefore, growth factors with fibrin-binding affinity are considered useful for tissue regeneration. Sakiyama-Elbert *et al*. produced a fusion protein between NGF and a short polypeptide of the α_2_-plasmin inhibitor (α_2_-PI) [25]; the activated coagulation factor XIII (FXIIIa, also known as plasma transglutaminase) mediated the cross-linking of this sequence to fibrin. In addition, a plasmin cleavage sequence was inserted between the α_2_-PI sequence and the growth factor. The resulting fusion protein (TG-P-NGF) was incorporated into fibrin clots by FXIIIa and was then released by plasmin when the tissues started to heal. TG-P-NGF was designed to be released “when cells demand.” The activity of the fusion protein was approximately 40% of that of native NGF, when assayed by neurite extension in PC12 cells. Unlike collagen-binding growth factors, this fusion protein bound to fibrin covalently. This could be related to the reduced activity observed in an *in vitro* assay.

The same or similar construct designs were applied to BMP2 [26], keratinocyte growth factor (KGF) [27], VEGF [28–30], ephrin B2 [31] and insulin-like-growth factor-I (IGF-I) [32]. The elevation of alkaline phosphatase activity in C3H10T1/2 cells mediated by the fibrin-binding BMP2 was similar to that observed for native BMP2, whereas its retention in a fibrin gel was more than that of the native protein (60% *vs*. 25% of added amount). The KGF fusion protein bound to fibrin gel and promoted the growth of epithelial cells after release from the gel. VEGF_121_ is a VEGF isoform that lacks the fibrin-binding sequence found in the major isoform (VEGF_165_) [30]. Fusion with the α_2_-PI sequence enabled the incorporation of VEGF_121_ into a fibrin gel. In addition, the fusion protein stimulated the growth of endothelial cells on the gel in a dose-dependent manner.

We produced another fibrin-binding growth factor: a chimeric protein of the fibronectin fibrin-binding domain (FBD) and EGF [33]. This fusion protein (FBD-EGF) bound to fibrin in the absence of the cross-linking enzyme (FXIIIa) and might be suitable for the preparation of fibrin-based scaffolds with EGF activity. The wound-healing potential of FBD-EGF was examined using an *in vitro* culture model of keratinocyte sheets; repair of the injured sheet was enhanced by fibrin-bound FBD-EGF but not by EGF alone.

### 3.3. Cell-Binding Fusion Proteins

Cell-binding growth factors have been designed to bind cell surface molecules such as integrins and receptors. The cell-binding domain of fibronectin (~30 kDa, including the RGD sequence that binds to integrins) was employed to produce EGF fusion protein (C-EGF) [34] or bFGF fusion protein (FN-FGF) [35]; proliferation of rat kidney fibroblasts or human umbilical vein endothelial cells was stimulated. However, their efficacy was not clearly shown. These fusion proteins might not be retained on the culture plates for long periods. An improved cell-binding EGF (containing an RGD sequence) was generated via the incorporation of hydrophobic linker sequences [36] and collagen-binding sequences [37] that ensured retention on culture substrate.

Van Lonkhuyzen *et al*. produced a chimeric protein of IGF-I and vitronectin (VN). This protein (VN: IGF-I) is associated with IGF-I-binding protein (IGF-BP) [38]. The complex of VN: IGF-I and IGF-BP bound to both the IGF-I receptor and to integrin, stimulated receptor-mediated and integrin-mediated pathways. As a result, the VN: IGF-I/IGF-BP complex exhibited a higher cell proliferation activity than the IGF-I/IGF-BP complex. Although the authors mainly emphasized the role of cross-talk in IGF-I receptor-mediated signaling and integrin-mediated signaling, vitronectin also seemed to contribute to cell adhesion via adsorption of the fusion protein to the substrate.

### 3.4. Organic Material-Binding Growth Factors

Growth factors have also been engineered to bind to artificial materials. Ogiwara *et al*. produced an EGF fusion protein coupled with the Fc region of immunoglobulin G (IgG) [39]. The Fc region is often used as a fusion tag to allow extracellular domains of large membrane proteins or receptor molecules to adhere nonspecifically to solid surfaces, thanks to its hydrophobicity [40]. Their fusion protein EGF-Fc was able to adsorb stably to tissue culture-treated surfaces, and mouse fibroblast Swiss 3T3 cells adhered to an EGF-Fc-coated surface. Phosphorylation of EGF receptors and the subsequent activation of mitogen-activated protein kinase (MAPK) were induced by the adsorbed EGF-Fc. MAPK activation was sustained even after 4 h, suggesting that EGF-Fc activated MAPK signaling continuously without internalization of the growth factor. The Fc region was also fused to leukemia inhibitory factor (LIF) [41]. Mouse embryonic stem cells maintained their undifferentiated state on LIF-Fc-coated surfaces. A fusion protein of Fc region and HGF also induced continuous activation of Akt signaling [42].

Artificial polypeptide sequences were also employed to generate binding growth factors. An elastinlike polypeptide consisting of (APGVGV)*_n_* or (GVGVP)*_n_* repeated sequences was used for binding to hydrophobic solid surfaces. The hydrophobicity of these peptide sequences could be altered in a thermosensitive manner; they showed hydrophilicity and detached from hydrophobic substrates below the phase transition temperature. Elloumi *et al*. produced fusion proteins of EGF and elastin-like peptide containing an RGD cell-binding sequence for enhancing cell adhesion [36,37]. Minato *et al*. fused an elastin-like peptide with IGF-binding protein 4 (IGF-BP4) and succeeded in promoting cardiomyocyte differentiation in mouse embryonic stem cells [43]. On the other hand, Doheny *et al*. fused the cellulose-binding domain of bacterial xylanase with the extracellular domain of stem cell factor (SCF) [44]. The fusion construct bound to cellulose tightly and stimulated the proliferation of SCF-dependent murine and human cells.

### 3.5. Titanium-Binding Growth Factors

Titanium is the metallic biomaterial most widely used for artificial joints and dental implants. Titanium is highly suitable for clinical use because of its high biocompatibility, good mechanical properties and excellent corrosion resistance. In addition, dental implants made of titanium can form tight adhesions between the titanium surface of implants and bone tissue (termed osseointegration). However, it takes as long as several weeks for osseointegration and patients have to wait for a longer period to complete recovery. Therefore, surface modification of titanium, including protein coating for improving biocompatibility and osseointegration is important to shorten the recovery period. However, it is generally difficult to modify the metal surface directly with biological molecules. To overcome this problem, high-throughput selection methods such as phage display, yeast display, ribosomal display or mRNA display have been developed. Phage display in particular was developed around 1990 and employed for the selection of peptides binding to inorganic substrates including BaTiO_3_ for electronic applications, SiO_2_, TiO_2_, aluminum, steel, semiconductors, platinum, silver and hydroxyapatite [45–60]. These are summarized in Table 2. Typically, phage display technologies introduce a combinatorial library (of the order of 10^9^ sequences) of 7-mer or 12-mer peptide sequences to a molecule, ligand or material. The technique has been utilized to identify amino acid sequences that recognize specific substrates, serving as a strategy to create biological linkers to bridge biomolecules and synthetic materials at the nanoscale. The selected peptides are called peptide aptamers (from “aptus” in Latin, the term was used for selected oligonucleotides for the first time).

Kashiwagi *et al*. fused BMP2 with an oligopeptide aptamer sequence to make Ti-binding peptide-1 (TBP-1) that has an affinity to titanium [61]. This peptide aptamer sequence was selected using a phage display system [58]. The fusion of this sequence allowed the reversible binding of BMP2 to titanium, with retention of its biological activity. However, they found that fusion of proteins with this peptide reduced its affinity to a TiO_2_ surface, and considered that intramolecular interactions between the peptide aptamer and the BMP moiety affected the binding affinity.

To avoid this problem, we recently constructed a new selection system consisting of a random peptide library fused with the EGF sequence to make a TiO_2_-binding EGF, using a ribosomal display system (unpublished). In this system, DNA sequences encoding both random sequence peptides and EGF were subjected to the selection system. Through this selection method, we expected to enhance the affinity to TiO_2_ surfaces modulated by intramolecular interactions between the growth factor and the binding portion. A peptide sequence fused with EGF (A8-EGF) was obtained after several rounds of selection, and its affinity to TiO_2_ surfaces and effects on cell proliferation were evaluated. The binding affinity of A8-EGF to TiO_2_ was higher than that of TBP-1 fused to EGF. This selection method appeared to reduce the possibility of conformational changes in binding growth factors, by fusing a random peptide library to growth factors ahead of selection. A8-EGF synthesized by a solid phase method also showed a high affinity to TiO_2_ after protein refolding. To evaluate the enhancement of cell proliferation, NIH3T3 cells were cultured in the presence of A8-EGF for two days and the rate of cell proliferation was estimated. A8-EGF itself enhanced cell proliferation as much as did unmodified EGF in the soluble state. This selection technology extends the possibility of designing binding growth factors.

## 4. Designs Based on Peptide Ligation

In parallel with progress in gene technology, chemical or enzymatic approaches for the synthesis of biological macromolecules have also progressed with the development of the Merrifield method [62]. The oligonucleotides and oligopeptides that are synthesized, have contributed significantly to biological and medical science and technology. Recent developments in the pharmaceutical industry require the large-scale production of biological macromolecules as drugs. However, although solidphase methods have realized the practical synthesis of oligonucleotides, oligopeptides and oligosaccharides, the chain lengths achieved have been limited. Therefore, similar to oligonucleotide ligation, some ligation methods including native chemical ligation have been developed for polypeptide chain extension to increase the chain length [63].

**Figure 5:**
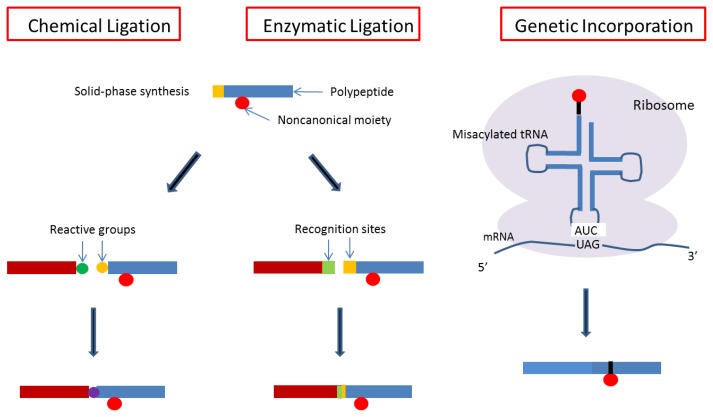
Methodology of bioorthogonal chemistry. Chemical ligation, enzymatic ligation, and genetic incorporation are included.

Ligation methods have also enabled us to synthesize more complicated macromolecules, and it has become a useful method in bioorthogonal chemistry, as shown in Figure 5 [64–70]. We can now prepare many types of complex macromolecules, including new synthetic polymer–biomacromolecular conjugates and biologically processed biomacromolecules. The former types include the conjugation of biological macromolecules with polyethylene glycol or stimuli-responsive polymers for medical or bioindustrial applications [71–77]. The latter molecules are biological modifications of DNA, RNA, or polypeptides. Therefore, it is now possible that growth factors and affinity peptides carrying noncanonical amino acids can be prepared separately and enzymatically conjugated into a single protein (Figure 3). To our knowledge, the design of a binding growth factor using this approach is the only example reported to date [78].

**Figure 6:**
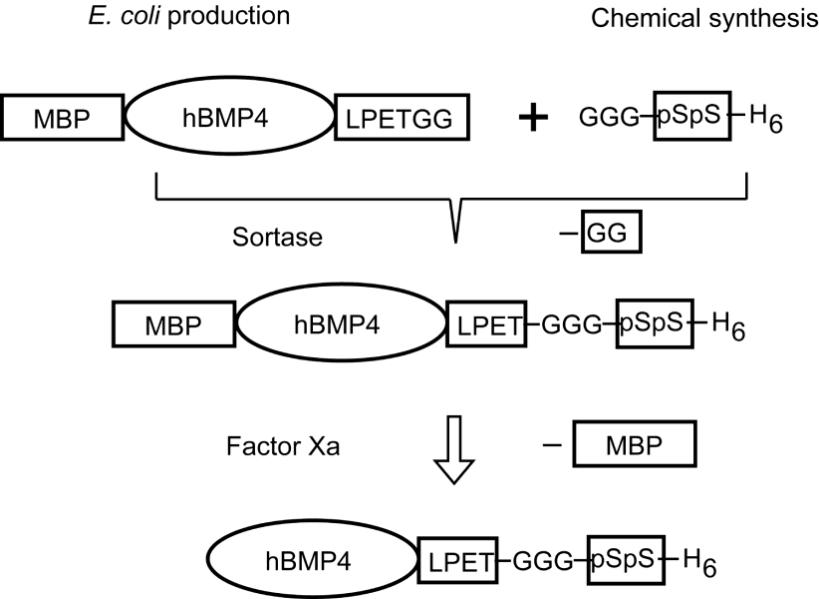
Scheme of the production and purification of hydroxyapatite-binding bone morphogenetic protein (BMP). Using a sortase reaction, a peptide carrying noncanonical amino acids (pSpS) was fused with the protein MBP-hBMP4 bearing a recognition sequence. After cleavage of the maltose-binding protein (MBP) moiety with Factor Xa, hBMP4-pSpS was isolated by His tag affinity.

One example is hydroxyapatite (HA) binding to BMP by conjugation with an active site of protein having affinity to tooth surfaces [78]. HA is well known as a bone implant material [79,80], because of its composition, similar to natural bone. The salivary gland protein statherin has an affinity to HA [81,82] and chemically synthesized short peptides designed from this protein have been reported [83]. In these, consecutive noncanonical amino acids—phosphorylated serines (pSpS)—were responsible for the affinity. Therefore, it was necessary to bind these noncanonical amino acids to BMP to prepare the HA-binding BMP. For this preparation, an enzymatic peptide ligation method was employed as shown in Figure 6. Soluble human BMP4 (hBMP4) with an enzymatic reaction target sequence at the *C*-terminal end was initially produced as fusion protein with maltose-binding protein (MBP) using an *Escherichia coli* expression system. For the enzymatic ligation, sortase A, a transpeptidase produced by *Staphylococcus aureus* to anchor cell surface proteins to the cell wall [84] was employed. The construct recognized short motifs of the amino acid sequence LPXTG at the *C*-terminal of one polypeptide and a GG sequence at the *N*-terminal of the other polypeptide. It then cleaved the peptide bond of the former peptide between its threonine and glycine followed by the formation of an amide bond to the latter peptide. This unique reaction has been utilized extensively for peptide ligation [85–87]. Because of the shortness of recognized peptide sequences, ligation by sortase A is likely to minimize the influence of the added sequences to any resulting proteins. After a peptide sequence containing phosphorylated serine (GGGpSpSH6) was synthesized chemically, these two polypeptides were ligated by sortase A reaction, and then the MBP moiety was removed by Factor Xa. The resulting conjugated growth factor (hBMP4-pSpS) was purified using a histidine tag. An hBMP4 conjugate containing canonical serines (hBMP4-SS) was prepared similarly to the control to compare the HA-binding affinity.

Both hBMP4-pSpS and hBMP4-SS bound to HA-beads in concentration-dependent manner as measured by an anti-BMP4 antibody binding within the range examined (0–2 μg/mL). The amount of bound hBMP4-pSpS was significantly higher than that of hBMP4-SS at each concentration examined. These data confirmed that the pSpS sequences were responsible for the enhanced HA binding of hBMP4-pSpS. To confirm the biological activity of the HA-bound BMP4 conjugate, multipotential C3H10T1/2 mesenchymal cells were seeded onto the HA beads with hBMP4-pSpS as described [88]. The expression levels of osteogenic genes (osteocalcin and osteopontin) known as markers of bone physiology [89] were enhanced in the cells after one week of culture. Protein-untreated beads and hBMP4-SS-treated beads induced osteocalcin and osteopontin expression within similar but lower ranges. This enhancement of the marker expression levels showed that hBMP4-pSpS retained authentic BMP4 activity after HA binding and that the increased binding of hBMP4-pSpS enhanced the osteogenic differentiation of C3H10T1/2 cells.

## 5. Application of Engineered Binding Growth Factors

The binding growth factors were designed to continue to stimulate signaling for a long time. This advantage was the result of substantial immobilization to natural tissues or to artificial scaffolds by simple addition. Therefore, their usefulness has been studied with regard to their efficacy in wound healing, tissue reconstruction and substitution.

### 5.1. Wound Healing

Collagen is exposed upon tissue injury. Thus, wound surfaces are considered to be good target sites for collagen-binding growth factors. CBD-EGF and CBD-HGF were retained on the wound surfaces and promoted closure in intractable skin wounds of diabetic (*db*/*db*) mice [22,90]. Implantation of a collagen sponge combined with CBD-EGF on skin wounds induced epithelialization above or underneath the sponge. Similar effects were observed for other collagen-binding factors such as bFGF (rhbFGF-F2, in collagen solution) [8] and PDGF-BB (combined with a collagen membrane) [91]. Fibrin-binding KGF (P-KGF) in combination with fibrin showed a potential for wound healing of human skin tissues grafted to athymic mice [27], although its efficacy was marginal compared with that of native KGF/fibrin. Collagen-binding growth factors were also applied to other wounded tissues, such as the colon destroyed by inflammation [9] and blood vessels injured and deprived of endothelial layer by a balloon catheter [90,92]. Restoration of the epithelial and endothelial layers was enhanced by the fused collagen-binding growth factors.

### 5.2. Repair of Cardiovascular Tissues by Implants

The angiogenic factors VEGF, bFGF and HGF have been studied for their therapeutic potential to restore the blood supply in ischemic tissues. The effects of gene-engineered constructs of these factors were also studied. Fibrin-binding VEGF_121_ (α_2_PI_1–8_-VEGF_121_) mixed with fibrin exhibited efficacious angiogenic properties. In a chorioallantoic membrane assay, the blood vessel density was ~1.5 times higher with fibrin-binding VEGF_121_ than that induced by native VEGF_121_. In addition, α_2_PI_1–8_-VEGF_121_ induced the formation of nonleaky vessels on a polytetrafluoroethylene chamber coated with fibrin and implanted subcutaneously [29].

Collagen-binding HGF (CBD-HGF) enhanced blood vessel migration into subcutaneously implanted collagen sponges that were combined with the fusion protein [23]. Endothelialization of the implanted collagen material prepared from heart valves was promoted when the material was combined with CBD-HGF (canine pulmonary artery implantation). Similar promotion was observed on polytetrafluoroethylene tubes coated with collagen and combined with CBD-HGF (*in vitro* culture). A sheet of collagen preparation (decellularized porcine urinary bladder matrix) could be combined with CBD-HGF, and angiogenesis was induced in injured tissues after implantation into mechanically injured porcine hearts [93].

### 5.3. Nerve Regeneration

Fibrin-binding NGF (TG-P-NGF) mixed with fibrin gel enhanced neurite extension from chick embryo dorsal root ganglia (organ culture) by 50% relative to native NGF and by 350% relative to the fibrin gel alone, in spite of the reduced activity of the fusion construct [25]. Collagen-binding NGF (CBD-NGF) combined with a collagen membrane and implanted subcutaneously enhanced nerve growth into the membrane [17]; the CBD-NGF-mediated nerve fiber density was approximately twice that observed for native NGF. It also enhanced healing of rabbit dermal ischemic ulcers.

### 5.4. Bone Regeneration

Demineralized bone matrix (DBM) combined with collagen-binding BMP2 (BMP2-h) was implanted subcutaneously in rats and induced ectopic bone formation [16,94]. BMP2-h-loaded DBM was also effective in the repair of rabbit mandibular bone defects, with 10–20% higher efficiency than that of native BMP2/DBM. In a rat craniotomy defect model, fibrin-binding BMP2 (TG-pI-BMP2) induced 76% more defect healing than native BMP2 [26]. This study was extended to the repair of long bones, as assessed by bone bridging.

## 6. Concluding Remarks

Growth factors that are able to bind to biological substances and artificial materials that can hardly retain native growth factors have been designed and synthesized. As a result, the stability of the resultant molecules was enhanced and the doses applied could be reduced. These considerations are also central to the production of biomaterials used as tissue repair scaffolds equipped with additional functions. There has been an increase in the number of reports on immobilized growth factors, since the effect of immobilized growth factors was first reported [95–101]. Thus, covalently immobilized growth factors have now been used effectively. The biological signals provided by immobilized growth factors last longer than with soluble ones. The long-lasting effect and the high local concentration on the surface, induced a significant biological effect on cells. Immobilization has been extended from organic materials to inorganic materials including metals and ceramics. However, chemical immobilization is insufficient for maximizing the efficacy of immobilized growth factors, because it is difficult to control their orientation. Therefore, to immobilize growth factors, genetic engineering and bioorthogonal chemistry approaches are required. These technologies enable specific modifications of growth factors, which can be immobilized at the desired orientations. This regulated immobilization enhances biological activity through specific interaction with cognate receptors of cells.

Considering recent progress in regenerative medicine, binding growth factors have become important molecules in culturing various kinds of stem cells and in constructing tissues. Additional properties for clinical use should be developed for binding growth factors, such as good stability in the human body and low immunogenicity.

## Figures and Tables

**Table 1:** Gene-engineered binding growth factors which bind to biological and organic materials.

Binding target		Growth factor	Fused polypeptide	Origin
Natural substrate	Collagen Gelatin	TGFβ1 [7,8], EGF [9], bFGF [10], BMP3 [11]	CBD polypeptide a (10 amino acids)	vWF
		EGF [12], bFGF [13]	CBD (20 kDa)	Bacterial collagenase
		PDGF [14], bFGF[15], BMP2 [16], NGF [17], BDNF [18], EGF [19], VEGF [20], NT3 [21]	CBD polypeptide b (7 amino acids)	Human collagenase
		EGF [22], HGF [23], VEGF [24]	CBD (40 or 27 kDa)	Fibronectin
	Fibrin Fibrinogen	NGF [25], BMP2 [26], KGF [27], VEGF [28,29,30], ephrin B2 [31], IGF-I [32]	FXIIIa substrate sequence c (8 or 12 amino acids)	α_2_-plasmin inhibitor
		EGF [33]	FBD (11 kDa)	Fibronectin
	Cell (integrin)	EGF [34], bFGF [35]	Cell-binding domain (30 kDa)	Fibronectin
		EGF [36,37]	Cell adhesive sequence d	Fibronectin
	Cell (integrin and IGF-I receptor)	IGF-I [38]	Vitronectin (full size)	Vitronectin
Artificial substrate	Solid surface	EGF [39,40], LIF [41], HGF [42]	Fc region	Immunoglobulin
		EGF [36,37], IGFBP4 [43]	Elastin-like polypeptide f (artificial)	Elastin
	Cellulose	SCF [44] (extracellular domain)	Cellulose-binding domain	Bacterial xylanase

Key: BDNF, brain-derived neurotrophic factor; bFGF, basic fibroblast growth factor; BMP, bone morphogenetic protein; CBD, collagen-binding sites or domain; EGF, epidermal growth factor; FBD, fibronectin fibrin-binding domain; FXIIIa, activated coagulation factor XIII; HGF, hepatocyte growth factor; IGF-I, insulin-like growth factor; IGF-IR, insulin-like growth factor-I receptor; IGFBP4, insulin-like growth factor-binding protein 4; KGF, keratinocyte growth factor; LIF, leukemia inhibitory factor; NGF, nerve growth factor; NT3, neurotrophin-3; PDGF-BB, platelet-derived growth factor BB (homodimer); SCF, stem cell factor; TGFβ, transforming growth factor β; VEGF, vascular endothelial growth factor; vWF, von Willebrand factor. Peptide sequences:

a WREPSFMALS;

b TKKTLRT;

c NQEQVSPL or LNQEQVSPRKKC;

d RGD;

e RKLPDA;

f (APGVGV)*_n_* or (GVGVP)*_n_*.

**Table 2:** List of inorganic material-binding peptides found by phage display.

Peptide sequence	Binding target	References
HQPANDPSWYTG/NTISGLRYAPHM	BaTiO_3_	46
CRRWESKRC	SiO_2_	47
CTKRNNKRC/CHKKPSKSC	TiO_2_	47
VPSSGPQDTRTT	Aluminum/steel	48
CNNPMHQNC/VISNHAESSRRL/SLTPLTTSHLRS	Semiconductor	49–51
PTSTGQA/CPTSTGQAC	Platinum	52
AYSSGAPPMPPF	Silver	53,54
YDSRSMRPH	ZnO	55
CGPRHTDGLRRIAARGPC	Cu_2_O	56
RRTVKHHVN	Fe_2_O_3_	57
RKLPDAPGMHTW	TiO_2_/Si/Ag	58
SVSVGMKPSPRP	Hydroxyapatite and tooth enamel	59
VTKHLNQISQSY	Hydroxyapatite and bone-like minerals	60
